# Drug-induced kidney stones: a real-world pharmacovigilance study using the FDA adverse event reporting system database

**DOI:** 10.3389/fphar.2025.1511115

**Published:** 2025-03-27

**Authors:** Pan Ding, Qinghua Luo, Leihua Cao

**Affiliations:** Department of urology, Nanchang People’s Hospital, Nanchang, China

**Keywords:** drugs, kidney Stones, FAERS, disproportionality analysis, pharmacovigilance

## Abstract

**Objective:**

This study aims to identify the drugs most commonly associated with kidney stone-related adverse events using data from the FDA Adverse Event Reporting System (FAERS), providing insights for clinical reference regarding the use of these drugs.

**Methods:**

We utilized the Medical Dictionary for Regulatory Activities (MedDRA 26.0) preferred term “nephrolithiasis” to identify drug-related adverse events (ADEs) for kidney stones reported in FAERS from Q1 2004 to Q1 2024. Reporting odds ratio (ROR) was used to quantify the signal strength of these ADEs, and new risk signals for kidney stones were compared with drug labeling information to identify any previously unreported risks.

**Results:**

Out of 21,035,995 adverse events reported in FAERS, 38,307 were associated with kidney stones. The top 5 drugs most frequently linked to kidney stone cases were adalimumab (2,636 cases), infliximab (1,266 cases), interferon beta-1a (920 cases), sodium oxybate (877 cases), and teriparatide (836 cases). Notably, certain drugs like lansoprazole (ROR 7.2, 95% CI 6.62–7.84), Xywav (ROR 7.1, 95% CI 6.03–8.35), and teduglutide (ROR 5.54, 95% CI 4.83–6.36) showed significant risk signals. Of the 50 drugs identified, 33 were not previously labeled as carrying a risk of kidney stones.

**Conclusion:**

Our analysis of FAERS data revealed new risk signals for kidney stones not indicated in the labels of 33 drugs. Close monitoring is recommended when using these medications, and further research is needed to investigate the mechanisms behind drug-induced kidney stone formation.

## 1 Introduction

Kidney stones are one of the most common diseases of the urinary system, with a rising incidence globally. The prevalence of kidney stones in the United States is approximately 10.1% (Abufaraj et al., 2021; Chewcharat and Curhan, 2021; Hill et al., 2022; Tundo et al., 2021), while in Europe it ranges between 5% and 10%, and in Asia, between 1% and 19% (Liyanage et al., 2022; Safdar et al., 2021; Thongprayoon et al., 2020). Additionally, the recurrence rate of kidney stones is notably high, with a 5-10 years recurrence rate of 50% and a 20-year recurrence rate of up to 75% (Jia et al., 2022; Ranjan et al., 2023; Soliman et al., 2017). Clinical studies have shown that approximately 80% of kidney stones are composed of a mixture of calcium oxalate and calcium phosphate, with uric acid and struvite stones accounting for 9% and 10%, respectively (Evan, 2010; Halinski et al., 2021; Nevo et al., 2020). Drug-induced kidney stones, though relatively rare, constitute only 1%-2% of cases (Christakos et al., 2019; Dobrek, 2020; Roedel et al., 2021) and are often overlooked due to the vast number of drugs involved. However, drug-induced kidney stones are largely preventable and can often be managed by discontinuing the offending drug.

The mechanism of drug-induced kidney stone formation primarily involves poor drug solubility, which leads to crystallization in the urine, or interference with the metabolism of calcium, oxalate, phosphate, uric acid, or purine, as well as alterations in urinary pH (Dobrek, 2020; Sutthimethakorn and Thongboonkerd, 2020; Wang H. et al., 2021). Currently, drug-induced kidney stones are mainly detected through specialized laboratory analyses of stone composition. However, the absence of information on a patient’s medication history in stone analysis can lead to an underestimation of the incidence of drug-induced stones. In 1980, Ettinger et al. conducted the first large-scale study on drug-induced kidney stones, finding that 0.4% of kidney stones in 50,000 U.S. cases contained triphenylmethane (Daudon et al., 2018; Ettinger et al., 1980). Subsequent research identified that protease inhibitors, sulfonamides, ceftriaxone, ephedrine-containing medications, calcium/vitamin D supplements, and carbonic anhydrase inhibitors can also induce stone formation (Blay et al., 2020; Daudon et al., 2018; Laditi et al., 2021; Laurence et al., 2020; Ma et al., 2021; Zhao and Angoff, 2019). With the continual development of clinical therapeutics, new research has shown that drugs such as benzopyridine, fructose, proton pump inhibitors, and low-dose aspirin may also contribute to kidney stone formation (Johnson et al., 2018; Pursnani and Streeper, 2024; Yang et al., 2023; Zhang et al., 2024). Urolithiasis and its associated complications pose significant health risks and adversely impact patients’ quality of life, while also placing a considerable burden on public healthcare systems (Li et al., 2023; Thongprayoon et al., 2020; Wang Q. et al., 2023). Therefore, it is imperative to pay close attention to drug-induced kidney stones in clinical settings and to identify potential drug-related risks in order to mitigate this complication.

The FAERS (FDA Adverse Event Reporting System) database is one of the largest voluntary reporting systems for adverse drug events worldwide, covering large-scale data from 2004 to the present. Maintained by the U.S. Food and Drug Administration (FDA), the database aims to monitor post-marketing drug safety. FAERS collects and analyzes reports of adverse drug events (ADEs) and medication errors to help identify potential drug safety issues. FAERS offers real-time updates, providing comprehensive data on adverse drug events, covering a wide range of drugs and patient populations, with high data transparency and accessibility (Cirmi et al., 2020; Grace et al., 2020; Wang et al., 2018). Its robust signal detection capabilities make it a critical tool in pharmacovigilance research, particularly in uncovering drug-related adverse events and evaluating drug safety. Although drug-induced kidney stones account for only 1%–2% of all kidney stone cases, their clinical impact is often underestimated. Many medications can contribute to kidney stone formation by altering urinary chemical composition or interfering with drug metabolism processes. Identifying these high-risk drugs and implementing appropriate preventive measures is crucial for reducing the incidence of drug-induced kidney stones. This study utilizes the FAERS database to systematically analyze adverse events related to kidney stones, revealing potential high-risk drugs and providing valuable references for clinical drug use.

## 2 Methods

### 2.1 Data source and extraction

The data for this study were sourced from the U.S. FDA Adverse Event Reporting System (FAERS) database, which collects a vast amount of information on adverse drug events (ADEs) and medication errors voluntarily submitted by healthcare professionals, manufacturers, and the public. We downloaded a total of 80 quarterly ASCII data packages from the FAERS database website, covering the period from 2004Q1 to 2024Q1 (https://open.fda.gov/apis/drug/event/). These data are organized into seven tables: demographic information (DEMO), adverse event records (REAC), drug usage records (DRUG), event outcomes (OUTC), adverse event outcomes (OUTC), sources of adverse events (PRSP), drug therapy duration (THER), and drug indications (INDI) (Banda et al., 2016; Iyer et al., 2014).

Given that FAERS data are based on spontaneous reporting, there is a risk of duplicate, withdrawn, or deleted reports. To ensure the accuracy of our study results, we followed the FDA’s recommended deduplication method. We selected the CASEID, FDA_DT, and PRIMARYID fields from the DEMO table and sorted the data by CASEID, FDA_DT, and PRIMARYID in that order. When duplicate CASEIDs were identified, the report with the most recent FDA_DT was retained. If both CASEID and FDA_DT were identical, the report with the largest PRIMARYID was kept (Jiang et al., 2014; Khaleel et al., 2022; Shu et al., 2022). This process was implemented using R software (version 4.3.1). After deduplication, each CASEID and PRIMARYID were unique, ensuring that only the most recent report for each patient was retained.

**FIGURE 1:**
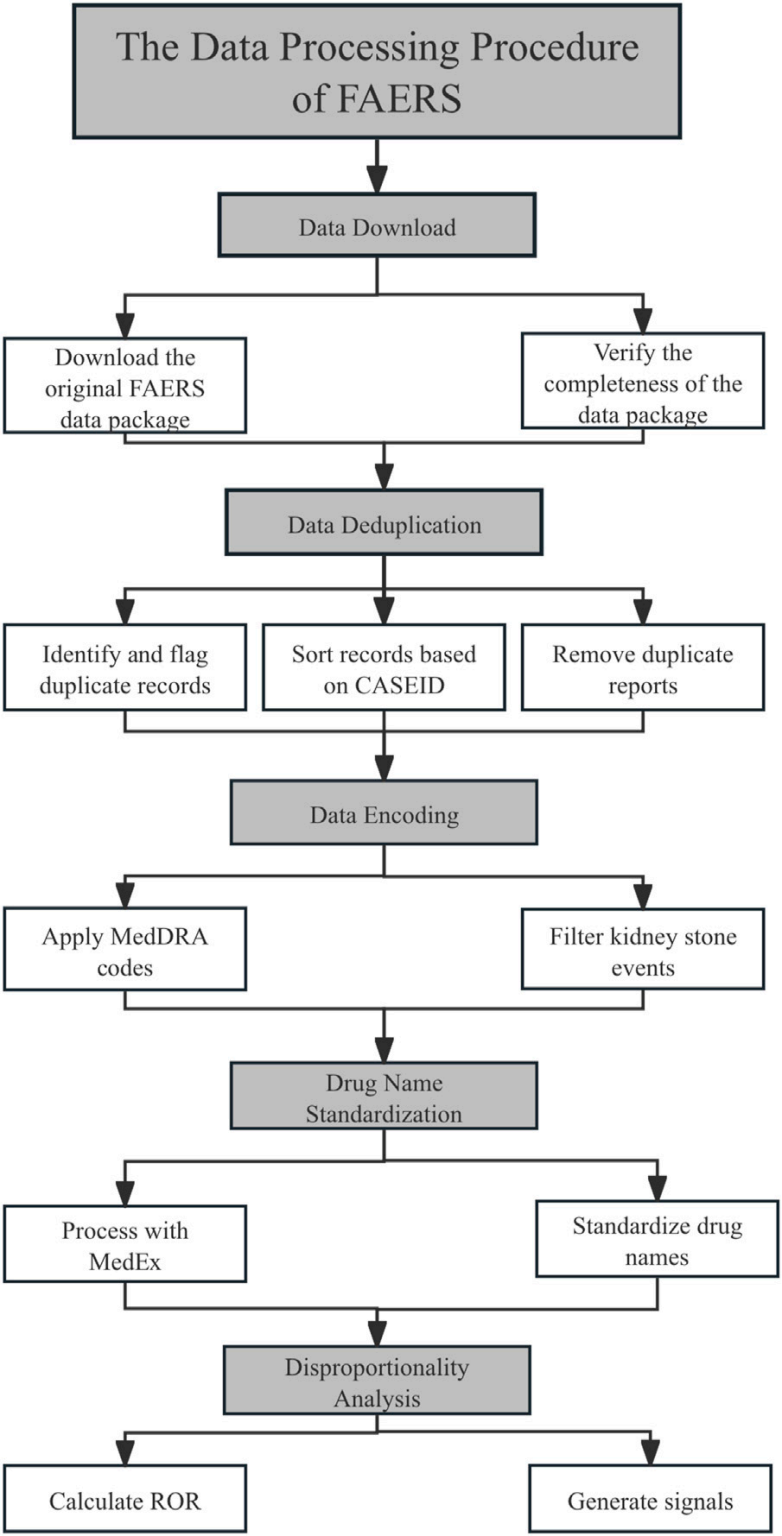
Flow chart for the identification of nephrolithiasis cases from the US Food and Drug Administration Adverse Event System (FARES).

All adverse event (ADE) data in the FAERS database are coded using Preferred Terms (PTs) from the Medical Dictionary for Regulatory Activities (MedDRA), with PTs being the most frequently used terms at the level 4 hierarchy in MedDRA. In this study, we used the PT “nephrolithiasis” (MedDRA version 26.0 code: 10029148) to extract relevant personal information, event outcomes, drug indications, and drug-related data for adverse event reports associated with kidney stones. We then used the generic name of the drug as the unique identifier for statistical analysis (Figure 1).

### 2.2 Drug definition

The drug names reported in the FAERS database lack standardization, as they are reported by a mix of healthcare professionals (e.g., physicians and pharmacists) and non-healthcare professionals (e.g., consumers and lawyers). This leads to a wide variety of drug names being reported. Simply using brand or generic names to search for drug-related adverse events poses a high risk of missing signals. To minimize this risk, we used the Medication Extraction System (MedEx), which has a name normalization accuracy of up to 97% (Jiang et al., 2014; Xu et al., 2010). We utilized the MedEx software (MedEx UIMA 1.3.8, Vanderbilt University, United States) to standardize drug names to their generic equivalents. Subsequently, we classified the top 50 drugs associated with kidney stones using the Anatomical Therapeutic Chemical (ATC) classification system (https://www.who.int/tools/atc-ddd-toolkit/atc-classification) endorsed by the World Health Organization. For drugs lacking a clear ATC code, we classified them according to their class or components listed in their product labels.

### 2.3 Disproportionality analysis

Disproportionality analysis is an important tool for evaluating potential causal relationships between drugs and adverse reactions. It is widely recommended for research using large databases (Barcelos et al., 2019; Beau-Lejdstrom et al., 2019; Caster et al., 2020; Khouri et al., 2021) Proportional Reporting Ratio (PRR), Reporting Odds Ratio (ROR), Information Component (IC), and Empirical Bayes Geometric Mean (EBGM) are four commonly used algorithms for signal detection in pharmacovigilance databases. Among these, ROR has demonstrated the best performance in pharmacovigilance studies (Bae et al., 2020; Hoffman et al., 2012; Ji et al., 2021; Lee et al., 2020). The ROR method is highly sensitive and can correct for many biases. Therefore, in this study, we used the ROR method to evaluate the top 50 drugs associated with kidney stones. This method compares the proportion of the target adverse event for a specific drug with the proportion for all other drugs to detect potential risk signals for kidney stone-inducing drugs (Huang et al., 2014). A positive signal is generated when a ≥3 and the lower limit of the 95% confidence interval (CI) of the ROR exceeds 1, indicating a statistically significant association between the drug and kidney stones (Supplementary Table S1). The higher the ROR value, the stronger the signal, suggesting a greater likelihood of a connection between the drug and kidney stones.

## 3 Results

### 3.1 Descriptive analysis

**FIGURE 2:**
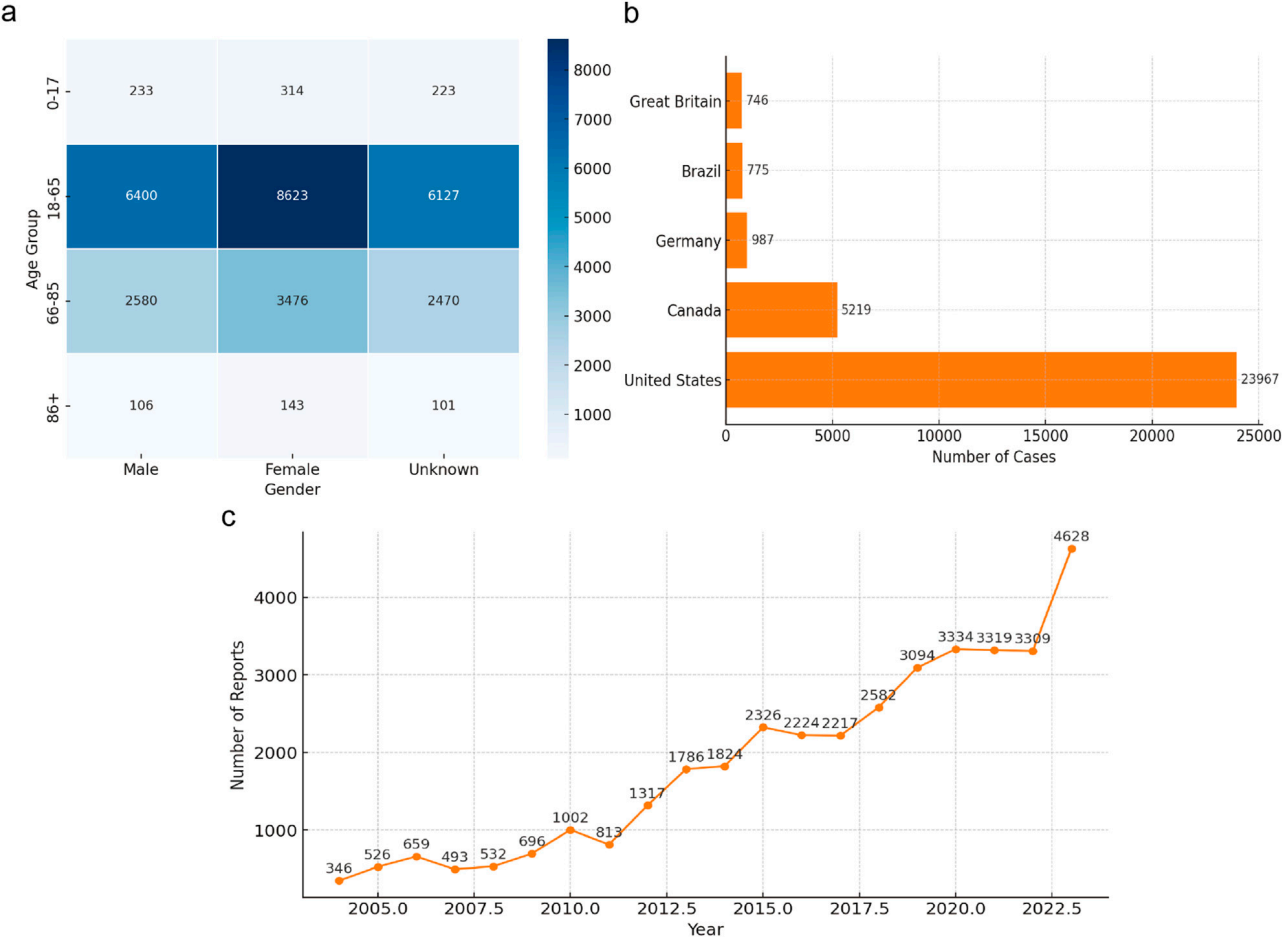
Clinical features of reported drug-induced nephrolithiasis. **(a)** Heatmap of Kidney Stone Cases by Age and Gender; **(b)** Geographic Distribution of Kidney Stone Cases; **(c)** Yearly Trend of Kidney Stone Reports (2004-2003).

From Q1 2004 to Q1 2024, the FAERS database reported a total of 2,103,599 adverse events (AEs). Among these, 38,307 cases were identified as kidney stones after data deduplication. The data were stratified by gender, age, reporting source, and country to explore the potential associations between patient characteristics and kidney stones. As shown in Figure 2, among the 38,307 drug-related kidney stone cases, the age group of 18-65 years accounted for the highest proportion, at 42.34% (approximately 16,219 cases), followed by the 66-85 age group at 17.07% (around 6,540 cases), those aged 17 and below at 1.54% (about 590 cases), and those aged 86 and above at 0.7% (around 268 cases). In terms of gender, female patients accounted for 53.16% of the total cases (approximately 20,365 cases), while males accounted for 39.46% (about 15,116 cases), and 37.77% of cases had unspecified gender. Kidney stone cases reported by pharmacists, physicians, and other healthcare professionals made up 11.35% of the total. Cases reported by consumers (18.79%) exceeded those reported by other sources. The country with the highest number of reported cases was the United States, with 14,339 cases (37.43%), followed by Canada (3,534 cases, 9.23%), Brazil (472 cases, 1.23%), the United Kingdom (394 cases, 1.03%), and Germany (372 cases, 0.97%).

From 2004 to 2023, the number of reported drug-induced kidney stone cases showed an upward trend, which may be related to the increased use of kidney stone-related drugs, heightened awareness of reporting adverse events associated with kidney stones, and advancements in detection technologies. Among all reports, 41.79% (approximately 16,078 cases) required hospitalization, 2.58% (about 987 cases) resulted in death, 1.68% (around 642 cases) were life-threatening, and 0.73% (about 280 cases) led to disability. Disproportionality Analysis.

### 3.2 Disproportionality analysis

To assess the potential association between drugs and kidney stones, a disproportionality analysis was conducted on the top 50 drugs ranked by the frequency of reports, using the Reporting Odds Ratio (ROR) to quantify the strength of the association. Supplementary Table S2 showed that the number of reported cases ranged from 145 to 2,636, with ROR values ranging from 0.54 to 33.29.

Among these drugs, adalimumab had the highest number of kidney stone-related reports, with 2,636 cases, followed by infliximab (1,266 cases), interferon beta-1a (920 cases), sodium oxybate (877 cases), and teriparatide (836 cases). In terms of signal strength, atazanavir had the highest ROR value (Figure 4), reaching 33.29 (95% CI 29.4–37.7), indicating a significant association with kidney stones. Other drugs with strong positive signals included topiramate (ROR 8.35, 95% CI 7.66–9.12), lansoprazole (ROR 7.20, 95% CI 6.62–7.84), Xywav (ROR 7.10, 95% CI 6.03–8.35), and teduglutide (ROR 5.54, 95% CI 4.83–6.36), which warrant clinical attention.

According to the Supplementary Table S2, the analysis further indicated that atazanavir exhibited the highest signal strength among all the drugs, suggesting a significant association with kidney stones. Additionally, Xywav, topiramate, and teriparatide also showed high ROR signals, indicating that these drugs may have a strong association with the occurrence of kidney stones.

**FIGURE 3:**
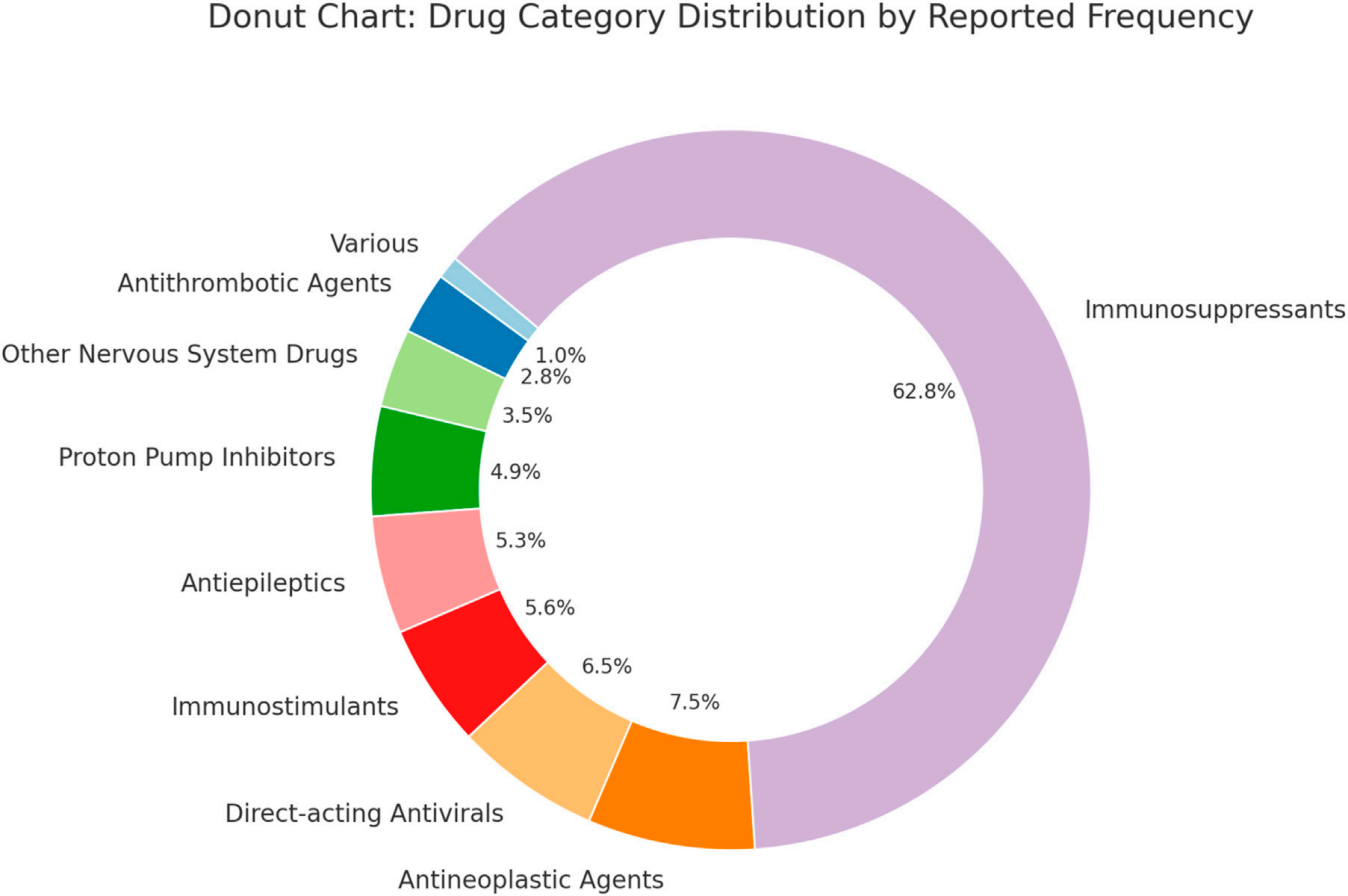
Classification of top 50 drugs associated with ADE of nephrolithiasis.

A further classification of the drugs revealed that, among the top 50 drugs associated with kidney stones, as shown in Figure 3, immunosuppressants accounted for 27.18%, making them the most common drug category, followed by antineoplastic agents (3.25%) and direct-acting antivirals (2.82%). A review of the most recent drug package inserts found that only nine drugs explicitly mentioned the risk of kidney stones, while the remaining 33 drugs, although meeting the signal detection criteria (≥3 cases and a lower limit of the 95% CI of the ROR greater than 1), did not have kidney stone risks listed in their package inserts. (Supplementary Table S3).

You may insert up to 5 heading levels into your manuscript as can be seen in “Styles” tab of this template. These formatting styles are meant as a guide, as long as the heading levels are clear, Frontiers style will be applied during typesetting.

## 4 Discussion

Our study identified and classified the drugs most closely associated with the induction of kidney stones in one of the largest adverse drug reaction reporting databases in the world, covering nearly 20 years of data. The proportion of drug-induced kidney stones has shown an increasing trend over the years, which warrants our attention. Additionally, this study is the first to use the FAERS database to reveal the potential association of 50 drugs with kidney stones, providing an important reference for pharmacovigilance and clinical risk management. In this study, we described the clinical characteristics of these adverse events and classified the drugs that induce kidney stones. Many of these drugs do not have kidney stone warnings in their package inserts, and their potential risk of inducing kidney stones is not widely recognized, highlighting them as potential kidney stone risk drugs.

The upward trend in reported kidney stone cases over the years, as depicted in Figure 2, may reflect increased drug usage, heightened awareness of adverse event reporting, and advancements in diagnostic technologies. This indicates that drug-induced kidney stones have become a significant clinical concern, necessitating enhanced monitoring and prevention efforts. Our age distribution analysis showed that the 18–65-year age group had the highest incidence of drug-induced kidney stones. Previous studies have found that kidney stone incidence peaks between 20 and 40 years of age (Tseng and Preminger, 2011; Tseng and Preminger, 2015), while others report a higher incidence in the 40 to 60 age group (Chewcharat and Curhan, 2021; Qiu F et al., 2021; Safdar OY et al., 2021). The higher incidence rate in the 18-65 age group may be related to the higher levels of drug exposure and metabolic activity in this age range. The decline in incidence in older age groups may be influenced by reduced drug use and physiological changes in metabolism (Chen et al., 2022; Ouyang et al., 2020). Further research is needed to understand how these factors influence kidney stone formation.

Notably, 41.79% of patients with drug-induced kidney stones required hospitalization (Figure 2), indicating a substantial impact on patient health and healthcare resources. Clinically, early identification and intervention—such as timely adjustment of medication regimens and enhanced patient education—are crucial for reducing hospitalization rates and improving outcomes.

Interestingly, as shown in Figure 2, our study found a higher incidence of drug-induced kidney stones in females, contrasting with previous research where males exhibited higher prevalence rates (Abufaraj et al., 2021; Chewcharat and Curhan, 2021; Tundo et al., 2021; Vicedo-Cabrera et al., 2020). This discrepancy may be due to differences in hormonal metabolism affecting stone formation, as androgens can increase urinary oxalate excretion by inhibiting osteopontin levels, leading to a higher incidence in males (Fuster et al., 2022; Xu et al., 2023; Zhu et al., 2019). However, our findings suggest that females may be more susceptible to drug-induced kidney stones. Factors such as increased use of certain immunosuppressants or hormonal medications among females could contribute to this risk (Abufaraj et al., 2021; Gillams et al., 2021; Knoll et al., 2022; Simonov et al., 2021). Further studies are needed to validate this finding and to explore the underlying mechanisms.

Geographically, the majority of reports originated from the United States (Figure 2), which could introduce reporting bias due to uneven distribution across regions. This necessitates cautious interpretation of the results and highlights the need for more balanced global reporting.

Overall, the increase in kidney stone reports, along with high hospitalization and mortality rates (Figure 2), indicates that drug-induced kidney stones significantly affect patient health and quality of life. Our demographic analysis suggests that individuals aged 18–65 years, female patients, and those in the United States are most affected. These findings underscore the importance of strengthening drug safety monitoring, conducting educational outreach, and raising awareness among both healthcare providers and patients regarding drug-induced kidney stones. Additionally, further research into the mechanisms of drug-induced kidney stone formation, particularly in high-risk populations, will aid in developing effective prevention and intervention strategies to reduce incidence and burden.

**FIGURE 4:**
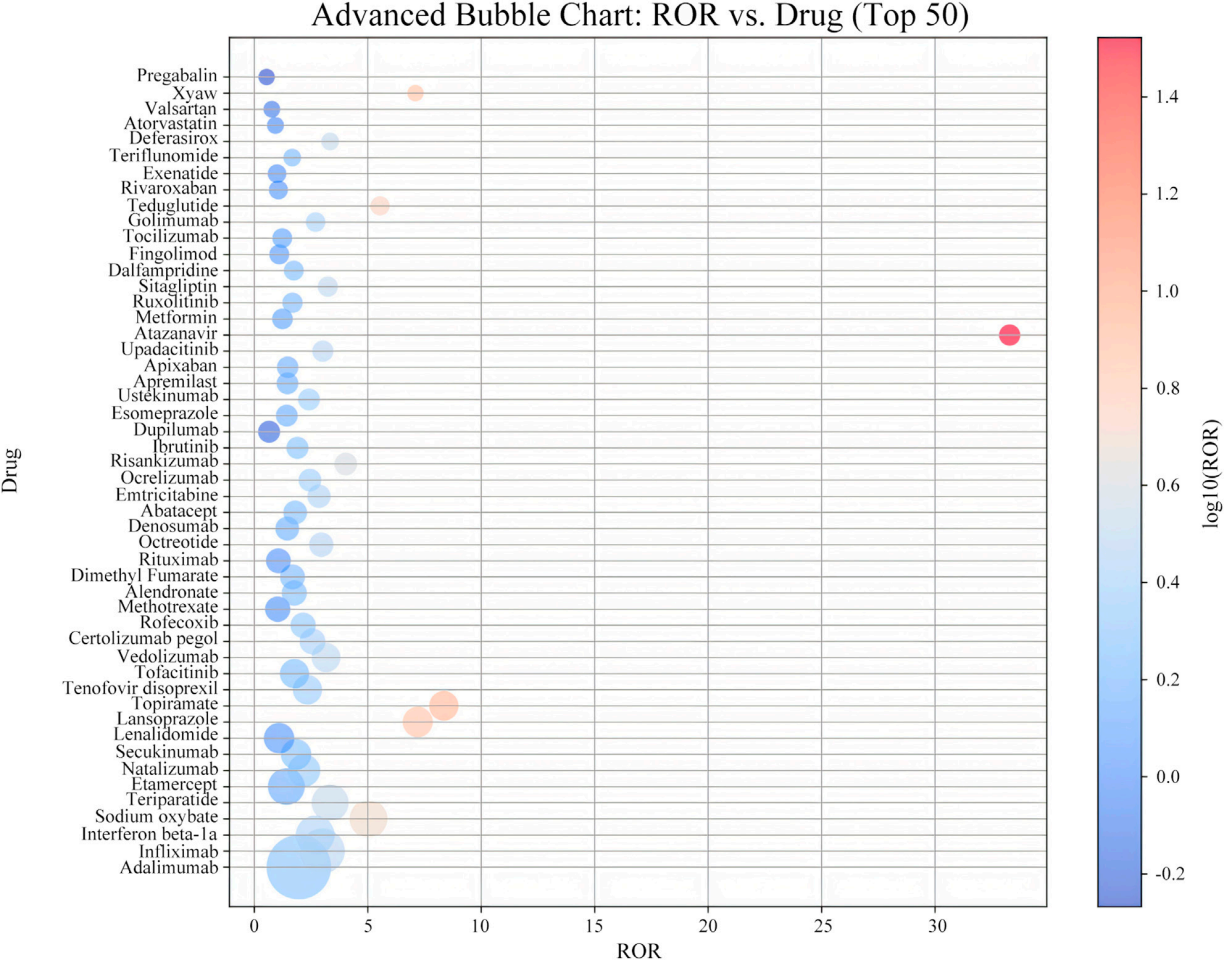
Distribution of ROR signal intensity associated with ADE of nephrolithia in the top 50 drugs.

Among the top 50 drugs with the highest frequency of kidney stone occurrence, only nine listed kidney stones as adverse reactions in their package inserts. Adalimumab had the highest number of cases (Figure 4); however, the mechanism by which it may cause kidney stones remains unclear and requires further study. Adalimumab is commonly used to treat inflammatory bowel disease (IBD) (Papamichael et al., 2019; Rakowsky et al., 2022; Tursi et al., 2023; Wang F. et al., 2023), and kidney stones are an extraintestinal manifestation of IBD, often presenting with urinary symptoms (Ganji-Arjenaki et al., 2017; Kumar et al., 2023; Rogler et al., 2021). Therefore, some kidney stone cases may be complications of IBD rather than direct adverse reactions to adalimumab, potentially leading to reporting bias.

Immunosuppressants are a high-risk category, possibly related to their metabolic pathways and immune-modulating effects. Antiviral and anticancer drugs show higher kidney stone risks due to their high urinary excretion rates. This is particularly relevant in patients undergoing long-term treatment with immunosuppressants or antivirals, where kidney function and urinary composition can be significantly altered, thereby increasing the likelihood of stone formation (Liu et al., 2020; Smith et al., 2021; Chen et al., 2019; Huang et al., 2022).

As illustrated in the Supplementary Table S3, atazanavir had the highest ROR value (33.29; 95% CI, 29.4–37.7), indicating the strongest association with kidney stones. Atazanavir, a protease inhibitor, induces kidney stone formation through crystallization due to poor urinary solubility. Although orally administered atazanavir is metabolized by the liver, approximately 7% is excreted unchanged by the kidneys. It has maximal solubility at a pH of 1.9, resulting in low solubility in urine and a propensity to precipitate as crystals (Brunel et al., 2019; Couzigou et al., 2007). Studies have shown that kidney stones generally appear after 2 years of atazanavir use, with chemical analyses revealing that stones consist of 40%–100% atazanavir without metabolites, sometimes mixed with calcium phosphate (Dhabliya et al., 2022; Plawecki et al., 2023; Santoriello et al., 2017). Most patients experience symptom relief upon discontinuation of the drug (de Lastours et al., 2013; Hamada et al., 2012). Recent studies (Singh et al., 2021; Harte et al., 2019) have further validated the crystallization risk of atazanavir in clinical use, emphasizing its stone formation mechanism in specific patient populations. Future research could involve in-depth exploration of the solubility changes of atazanavir in different pH environments to further validate its crystallization mechanism and investigate therapeutic strategies to improve solubility and reduce the risk of stone formation.

Topiramate had the second-highest ROR value. Unlike atazanavir, topiramate induces kidney stones by inhibiting carbonic anhydrase in renal tubules, leading to renal tubular acidosis, decreased plasma bicarbonate concentration, and urine alkalization, which promote calcium phosphate stone formation (Barnett et al., 2018; Daudon et al., 2018). Recent studies (Wang Z. et al., 2021; Zhang et al., 2020) have further validated the mechanism of topiramate-induced kidney stones, especially with long-term use, where the correlation between urine alkalinization and stone formation becomes more pronounced. Understanding the composition of kidney stones and the metabolic effects of medications can aid in exploring the mechanisms by which drugs induce kidney stones and in developing preventive strategies. Future research could analyze the specific effects of topiramate use at different doses and treatment durations on urine pH and stone formation, to verify the causal relationship between urine alkalinization and stone risk (Roh et al., 2018; Lo et al., 2020).

Furthermore, among the top 50 drugs not listing kidney stone risks in their labels, 33 met the signal detection criteria—having at least three reported cases and a lower limit of the 95% CI of the ROR greater than 1. This suggests potential new risk signals for kidney stones associated with these drugs, warranting attention. For example, previous reports indicated that an HIV-infected individual developed kidney stones and hydronephrosis after initiating tenofovir-containing highly active antiretroviral therapy (HAART) (Cicconi et al., 2004; Mangal et al., 2022). Given that tenofovir is primarily excreted by the kidneys, its association with kidney stones merits further investigation.

Zakaria et al. found an increased risk of kidney stones in patients using vedolizumab (Alameddine et al., 2023). Compared to non-biologic therapies, the combination of two or more immunobiologics also increased the risk of kidney stones. Our study similarly highlights immunosuppressants as the most significant category in drug-induced kidney stones, reinforcing their crucial role. Deferasirox, used for treating thalassemia, has been associated with high incidences of hypercalciuria and kidney stone formation (Aliberti et al., 2022; Capolongo et al., 2020).

Conversely, some drugs may have protective effects against kidney stones. For instance, Hiroya et al. found that alendronate sodium inhibits calcium stone formation, suggesting it may prevent urolithiasis rather than promote it (Senzaki et al., 2004). Animal studies have shown that atorvastatin can inhibit kidney stone formation (Liu et al., 2022; Tsujihata et al., 2008). In our analysis, atorvastatin’s ROR value was close to 1, indicating no significant association, which could be due to differences in research methods or sample characteristics. Since disproportionality analysis only suggests associations rather than causal relationships, further mechanistic studies and clinical validations are necessary.

It is important to note that disproportionality analysis only suggests an association between a drug and an adverse event, but does not establish causality. The voluntary nature of FAERS reporting may lead to either underestimation or overestimation of risks. Additionally, regional biases in the data should be interpreted with caution. To further clarify the relationship between drugs and kidney stones, more mechanistic studies and clinical validations are needed.

This study provides valuable insights into pharmacovigilance, particularly regarding drugs that may contribute to kidney stone formation. However, several limitations exist. First, The voluntary reporting mechanism of the FAERS database may lead to incomplete or biased data. Second, most reports originate from the United States, with relatively few from other countries, potentially leading to statistical bias. Additionally, The signal detection method used in this study can only reveal associations, not establish causality. Future research could combine prospective clinical trials and biological mechanism studies to validate the causality of high-signal drugs. Moreover, future research should focus on the long-term effects of different drug combinations and the comprehensive impact of metabolic factors on kidney stone risk.

## 5 Conclusion

From the FAERS database reports spanning from the first quarter of 2004 to the first quarter of 2024, we identified 50 high-signal drugs associated with kidney stones, 33 of which were not listed as a risk on the drug labels. This provides important insights for drug risk assessment and clinical intervention. Given that drug-induced kidney stones may lead to decreased adherence to primary medications, clinicians should carefully evaluate the risks when prescribing these drugs, particularly for high-risk populations. Our list of drugs can serve as a valuable reference for clinicians, assisting them in selecting more appropriate medications for patients at high risk of kidney stones, thereby reducing the incidence of kidney stones and improving patient outcomes. Future research should combine biological mechanism validation, prospective studies, and global collaborative databases to further explore the causality of drug-induced kidney stones and potential intervention strategies.

## Data Availability

Publicly available datasets were analyzed in this study. This data can be found here: https://open.fda.gov/apis/drug/event/.
