# Supplementary material for: Drug-induced kidney stones: a real-world pharmacovigilance study using the FDA adverse event reporting system database
Source: Front Pharmacol. 2025 Mar 27;16:1511115. doi: 10.3389/fphar.2025.1511115 (PMC11983471; doi:10.3389/fphar.2025.1511115)
Supplement: Supplementary file 3 [file Table1.docx]

Supplementary Table S1. Summary of algorithms used for signal detection.

| Algorithms | Equation | Criteria |
| --- | --- | --- |
| ROR | ROR = ad/bc  95%CI = eln(ROR)±1 .96(1/a+1/b+1/c+1/d)∧0 . 5 | lower limit of 95% CI > 1, n≥ 3 |

a, number of reports containing both the suspect drug and the suspect adverse drug reaction; b, number of reports containing the suspect adverse drug reaction with other medications (except the drug of interest); c, number of reports containing the suspect drug with other adverse drug reactions (except the event of interest); d, number of reports containing other medications and other adverse drug reactions. ROR, reporting odds ratio;
